# Synthesis of Tin-Doped Three-Dimensional Flower-like Bismuth Tungstate with Enhanced Photocatalytic Activity

**DOI:** 10.3390/ijms23158422

**Published:** 2022-07-29

**Authors:** Xiaodong Zhu, Fengqiu Qin, Xiuping Zhang, Yuanyuan Zhong, Juan Wang, Yu Jiao, Yuhao Luo, Wei Feng

**Affiliations:** 1 School of Mechanical Engineering, Chengdu University, Chengdu 610106, China; xiaodangjia21@126.com (X.Z.); mysumeiren@163.com (F.Q.); 18781235109@163.com (X.Z.); suzyy605@163.com (Y.Z.); wangjuan9760@163.com (J.W.); 2 School of Science, Xichang University, Xichang 615013, China; 3 College of Materials and Chemistry & Chemiacl Engineering, Chengdu University of Technology, Chengdu 610051, China; lyh1609460550@163.com

**Keywords:** Bi_2_WO_6_, Sn doping, 3D flower-like, photocatalytic activity, hydrothermal method

## Abstract

Photocatalytic degradation of harmful organic matter is a feasible and environmentally friendly method. Bi_2_WO_6_ has become a hotspot of photocatalysts because of its unique layered structure and visible light response. In the present study, Sn doping was adopted to modified Bi_2_WO_6_ by hydrothermal method. The Sn-doped Bi_2_WO_6_ photocatalysts were characterized by XRD, SEM, TEM, BET, XPS, PL, and DRS, respectively. The results show that Sn-doped Bi_2_WO_6_ shows three-dimensional (3D) flower-like morphology, which is composed of two-dimensional (2D) nanosheets. Sn^4+^ ions enter into the Bi_2_WO_6_ lattice, producing a degree of Bi_2_WO_6_ lattice distortion, which is in favor of reducing the recombination of photogenerated electrons and holes. Moreover, the specific surface area of Bi_2_WO_6_ is significantly increased after doping, which is beneficial to providing more active sites. The photocatalytic results show that 2%Sn-Bi_2_WO_6_ exhibits the highest photocatalytic activity. After 60 min of irradiation, the photocatalytic degradation degree of methylene blue (MB) increases from 80.6% for pure Bi_2_WO_6_ to 92.0% for 2%Sn-Bi_2_WO_6_. The first-order reaction rate constant of 2%Sn-Bi_2_WO_6_ is 0.030 min^−1^, which is 1.7 times than that of pure Bi_2_WO_6_.

## 1. Introduction

With the rapid development of economy and society, the problem of water pollution has become increasingly prominent [1,2,3]. Semiconductor photocatalysis degradation technology is widely studied as it is a green and feasible way for environmental protection [4,5,6]. Traditional semiconductor photocatalysts (TiO_2_, ZnO, CeO_2_, etc.) usually only absorb ultraviolet light, but ultraviolet light accounts for less than 5% of sunlight, which greatly limits the utilization efficiency of solar energy [7,8,9,10]. Researchers have developed several high-efficiency visible light response photocatalysts to improve the utilization of sunlight [11,12,13]. Bi_2_WO_6_ has attracted much attention due to its unique layered structure and good visible light response [14,15,16]. Sharma [16] et al. prepared 2D Bi_2_WO_6_ nanosheets by hydrothermal method, which shows a band gap of 2.78 eV. The degradation degree of ciprofloxacin is 47% under 150 min natural light irradiation. However, in practical applications, the photocatalytic activity of pure Bi_2_WO_6_ is greatly limited due to its rapid recombination of photogenerated electrons and holes. Employing metal ion doping to modify Bi_2_WO_6_ is one of the methods used to enhance the photocatalytic activity [17,18,19]. On one hand, new impurity levels will be formed by cation doping, which reduces the excitation energy of transition [20,21]. On the other hand, ion doping will produce lattice distortion, trapping photogenerated charges and improving the separation of carriers [22,23].

Generally, photocatalysts that present three-dimensional (3D) morphology exhibit higher photocatalytic activity than other morphology as 3D morphology is beneficial to providing more reactive active sites due to its larger specific surface area and the utilization of visible light owing to the multiple reflection of light in 3D structures [24,25,26]. In our previous work, the 3D flower-like Bi_2_WO_6_ photocatalysts were prepared and it was found that the optimum hydrothermal temperature is 160 °C [27]. On this basis, in the present work, to improve the photogenerated charge separation and specific surface area of pure Bi_2_WO_6_, Sn doping modification was adopted and Sn-doped Bi_2_WO_6_ photocatalyst was prepared under the hydrothermal conditions of 160 °C and 12 h. The crystal structure, surface morphology, specific surface area, elemental composition and valence state, and optical properties of samples were analyzed using various characterization methods. The photocatalytic activity of samples was evaluated by the degradation of methylene blue (MB), and the mechanism of Sn doping improving the photocatalytic activity was comprehensively analyzed.

## 2. Results and Discussion

### 2.1. Catalyst Characterization

**Figure 1:**
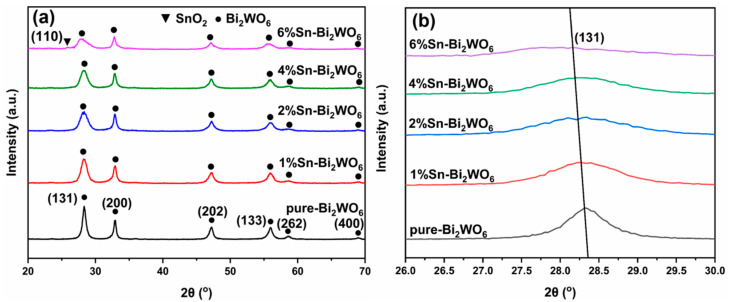
The XRD patterns of samples (**a**) and the peak of (131) crystal plane (**b**).

Figure 1a shows the XRD patterns of pure Bi_2_WO_6_ and Sn-Bi_2_WO_6_. The diffraction peaks appear at 28.3°, 32.8°, 47.1°, 56.0°, 58.5°, and 68.8°, corresponding to the (131), (200), (202), (133), (262), and (400) crystal planes of Bi_2_WO_6_ [15,17,18]. The Sn-Bi_2_WO_6_ patterns show that the shape and position of the diffraction peaks are similar to pure Bi_2_WO_6_. No diffraction peak related to Sn is detected in the patterns of 1%Sn-Bi_2_WO_6_, 2%Sn-Bi_2_WO_6_ and 4%Sn-Bi_2_WO_6_. A part of Sn^4+^ ions can enter the Bi_2_WO_6_ lattice to replace Bi^3+^ without reacting with Bi_2_WO_6_ to generate a new phase [18,23]. Lattice expansion or shrinkage caused by ion doping is controversial. Generally, when ions with a radius less than Bi^3+^ enter the lattice and replace them, the lattice will shrink. According to the Bragg equation, the XRD diffraction peak will shift to higher angle [18,21]. On the contrary, some research shows that the XRD diffraction peak shifts to a smaller angle after ions with an ionic radius less than Bi^3+^ doping, indicating that it causes lattice expansion [20,28]. In the study of Sm-doped Bi_2_WO_6_ reported by Liu et al. [20], it was found that when Sm^3+^ ions with an atomic radius (0.096 nm) less than Bi^3+^ (0.108 nm) were doped, the XRD diffraction peaks shifts to a lower angle, indicating that the lattice expansion is caused after doping. They believe that after Sm^3+^ is doped, part of Sm^3+^ will replace Bi in its lattice, and part of Sm^3+^ will enter the interstitial position of the Bi_2_WO_6_ lattice, causing lattice expansion. Alhadi et al. [28] believe that the same phenomenon appears in their Sn-doped Bi_2_WO_6_ research. In this work, it can be seen from the enlarged XRD pattern (Figure 1b) that the diffraction peak of the (131) crystal plane shifts slightly to a small angle after Sn doping, which indicates that the lattice of Bi_2_WO_6_ is expanding. The lattice parameters and cell volumes were calculated, as shown in Table 1. The results show that the lattice expands slightly after Sn doping, which is consistent with the literature [20,28]. Because the ion radius of Sn^4+^ (0.069 nm) [28] is smaller than Bi^3+^ (0.108 nm) [20], the lattice will shrink when it replaces Bi^3+^. However, lattice expansion occurred in this study, which may be due to the fact that in addition to the Sn^4+^ ions replacing Bi^3+^, the remaining Sn^4+^ ions will enter the interstitial position of the Bi_2_WO_6_ lattice, causing lattice expansion. Moreover, there is a view that the change in electronic environment will also cause lattice changes. Chen et al. [29] believe that when elements with large electronegativity are used to replace elements with small electronegativity, the mutual attraction between atoms will be enhanced, which will cause lattice contraction. Replacing elements with small electronegativity with elements with large electronegativity will weaken the mutual attraction between atoms and cause lattice expansion. In this study, since the electronegativity of Sn (Pauling, 1.96) [30] is lower than that of Bi (Pauling, 2.02) [31], when Sn^4+^ replaces Bi^3+^, the attractive force between Sn atoms and O, W atoms will be reduced, resulting in lattice expansion.

Compared with the diffraction peak of pure Bi_2_WO_6_, the wider FWHM of the diffraction peak and the lower intensity of the diffraction peak imply that the grain size decreases and the amorphous composition increases after Sn doping [28]. Bi-O-Sn bonds will be formed by Sn doping, which hinders the migration of Bi, O, and W atoms, delaying the nucleation and growth of Bi_2_WO_6_ [32]. The crystallite size of samples can be calculated using the Scherrer formula [22,33]. The grain sizes of pure Bi_2_WO_6_, 1%Sn-Bi_2_WO_6_, 2%Sn-Bi_2_WO_6_, 4%Sn-Bi_2_WO_6_, and 6%Sn-Bi_2_WO_6_ are 12.2, 7.4, 6.0, 6.1, and 4.7 nm, respectively, indicating that the grains are refined by Sn doping. The crystallinities of pure Bi_2_WO_6_, 1%Sn-Bi_2_WO_6_, 2%Sn-Bi_2_WO_6_, 4%Sn-Bi_2_WO_6_, and 6%Sn-Bi_2_WO_6_ are 90.8%, 88.9%, 87.7%, 86.3%, and 79.0%, respectively. The crystallinity of Sn-Bi_2_WO_6_ decreases gradually with the increase in Sn doping concentration. Remarkably, there is a diffraction peak near 26.8° in the pattern of the 6%Sn-Bi_2_WO_6_ sample, which corresponds to the SnO_2_ (110) crystal plane, which indicates that SnO_2_/Bi_2_WO_6_ composite photocatalyst forms at a high Sn doping concentration due to the excess of Sn^4+^ in the Bi_2_WO_6_ lattice solubility, forming a new phase SnO_2_ [33,34].

**Figure 2:**
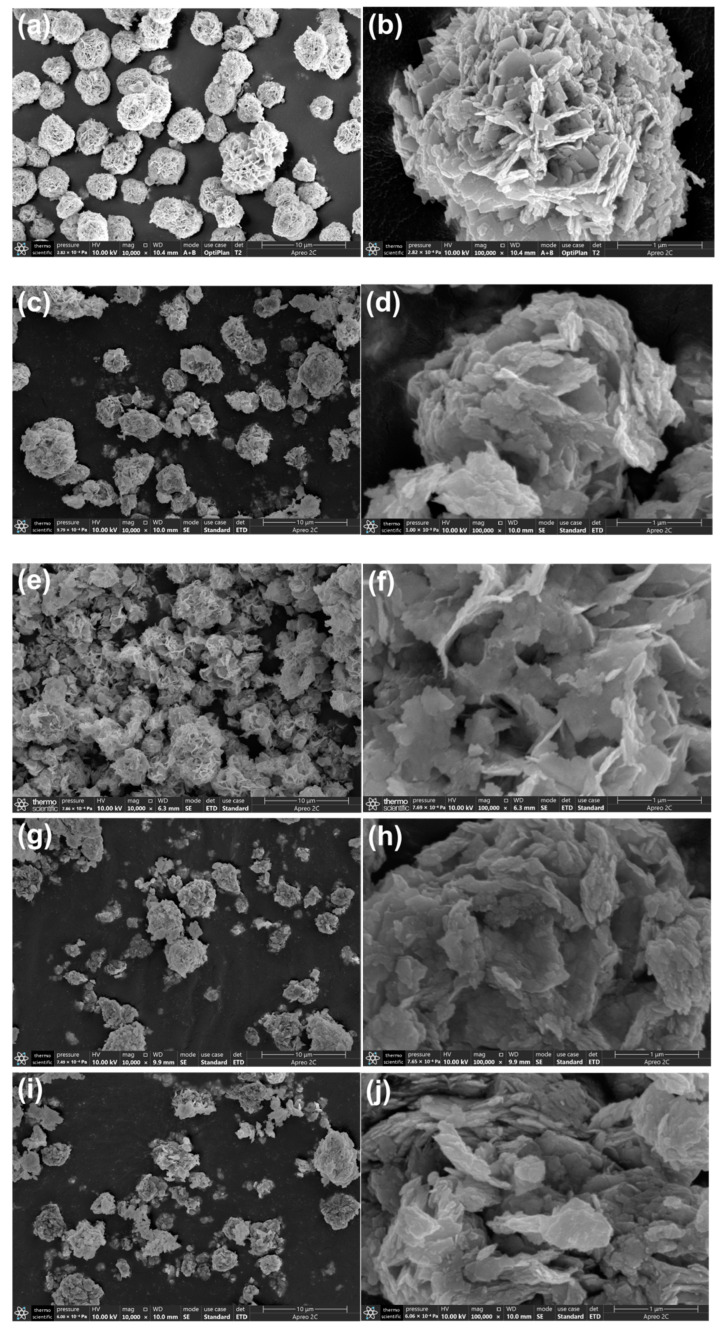

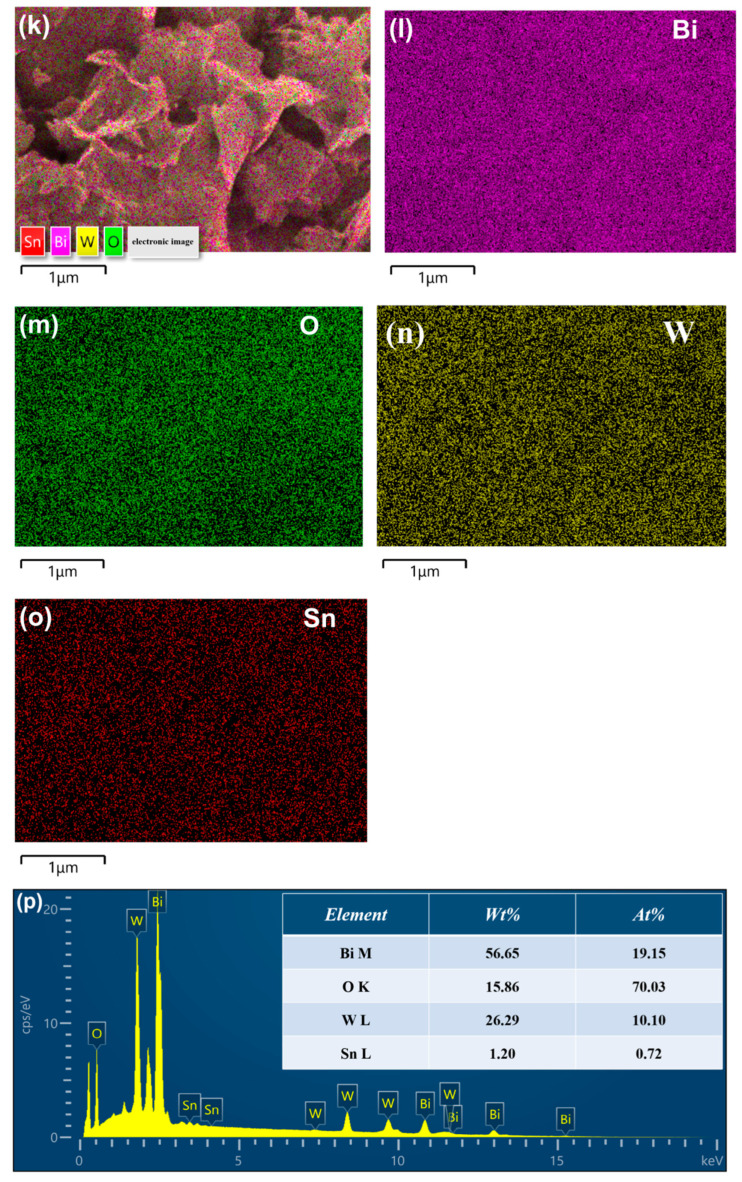
SEM images of pure Bi_2_WO_6_ (**a**,**b**), 1%Sn-Bi_2_WO_6_ (**c**,**d**), 2%Sn-Bi_2_WO_6_ (**e**,**f**), 4%Sn-Bi_2_WO_6_ (**g**,**h**), and 6%Sn-Bi_2_WO_6_ (**i**,**j**), element mappings of Bi, O, W, Sn (**k**–**o**), and EDS analysis of 2%Sn-Bi_2_WO_6_ (**p**).

The SEM images of samples are shown in Figure 2a–j. In Figure 2a,b, it can be clearly observed that Bi_2_WO_6_ is composed of 2D nanoflakes interwoven with each other in the shape of 3D flower-like with an average diameter of 2–4 μm, and the length of the nanoflakes varies in size from a few tens to hundreds of nanometers. It is observed in Figure 2c–j that 3D morphology of Sn-Bi_2_WO_6_ is similar to Bi_2_WO_6_, and the length of 2D nanosheets is concentrated at 0.5–1 μm. Remarkably, the flake thickness of Sn-Bi_2_WO_6_ decreases, which is conducive to increasing the surface area. Figure 2k–p shows the element mappings of 2%Sn-Bi_2_WO_6_, from which it is known that 2%Sn-Bi_2_WO_6_ contains Bi, O, W, and Sn elements, and these elements are uniformly distributed. Figure S1 shows the EDS results of 1%Sn-Bi_2_WO_6_ (a), 4%Sn-Bi_2_WO_6_ (b), and 6%Sn-Bi_2_WO_6_ (c). The actual Sn/Bi molar ratio in the doped samples was measured. The molar ratios of Sn/Bi in 1%Sn-Bi_2_WO_6_, 2%Sn-Bi_2_WO_6_, 4%Sn-Bi_2_WO_6_, and 6%Sn-Bi_2_WO_6_ are 1.5%, 3.8%, 5.2%, and 6.6%, respectively. With the increasing Sn concentration, the Sn/Bi ratio shows an upward trend.

**Figure 3:**
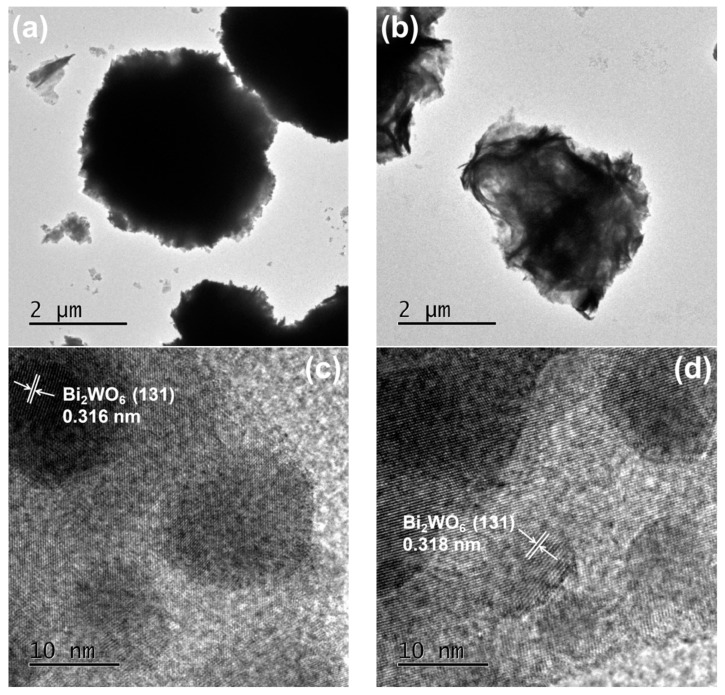
TEM images of pure Bi_2_WO_6_ (**a**) and 2%Sn-Bi_2_WO_6_ (**b**), HRTEM images of pure Bi_2_WO_6_ (**c**) and 2%Sn-Bi_2_WO_6_ (**d**).

Figure 3 shows the TEM images of Bi_2_WO_6_ (a) and 2%Sn-Bi_2_WO_6_ (b). It can be seen that the three-dimensional morphology of Bi_2_WO_6_ is nearly a regular sphere, with a diameter of 3 μm (Figure 3a). After Sn doping, the diameter of the sample does not change obviously. According to HRTEM images of samples, the interplanar spacing of Bi_2_WO_6_ is about 0.316 nm (Figure 3c), which basically agrees with the theoretical value of the (131) plane of Bi_2_WO_6_. In Figure 3d, the interplanar spacing 0.318 nm is observed in 2%Sn-Bi_2_WO_6_, which corresponds to the (131) plane [35].

**Figure 4:**
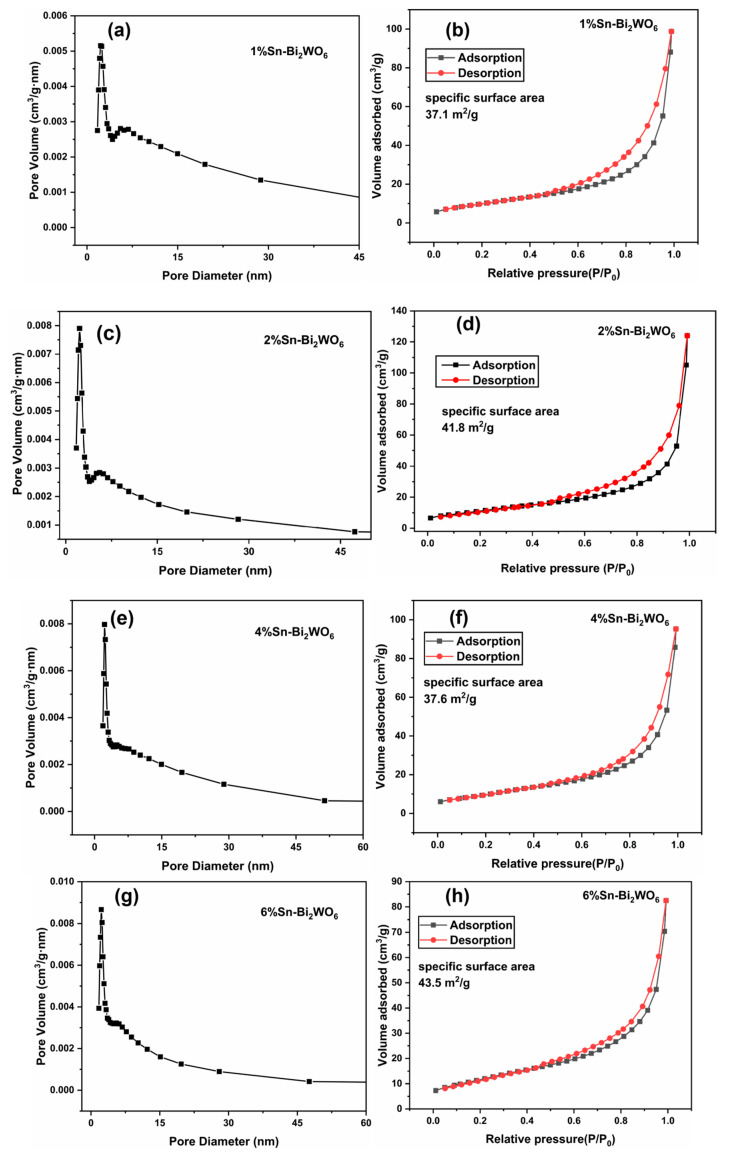
Pore size distribution curves and N_2_ adsorption–desorption isotherms of 1%Sn-Bi_2_WO_6_ (**a**,**b**), 2%Sn-Bi_2_WO_6_ (**c**,**d**), 4%Sn-Bi_2_WO_6_ (**e**,**f**), and 6%Sn-Bi_2_WO_6_ (**g**,**h**).

Figure 4 shows the pore size distribution curves and the N_2_ adsorption–desorption isotherms of Sn-Bi_2_WO_6_. The pore size of pure Bi_2_WO_6_ is mainly concentrated at 0–30 nm [27], while the pore sizes of Sn-Bi_2_WO_6_ are mainly concentrated at 0–15 nm. The specific surface areas of 1%Sn-Bi_2_WO_6_, 2%Sn-Bi_2_WO_6_, 4%Sn-Bi_2_WO_6_, and 6%Sn-Bi_2_WO_6_ are 37.1, 41.8, 37.6, and 43.5 m^2^/g, respectively. The specific areas of Sn-doped Bi_2_WO_6_ are significantly higher than pure Bi_2_WO_6_ (20.8 m^2^/g) [27]. Sn doping results in smaller grain sizes and thinner nanosheets than pure Bi_2_WO_6_, increasing the specific surface area.

**Figure 5:**
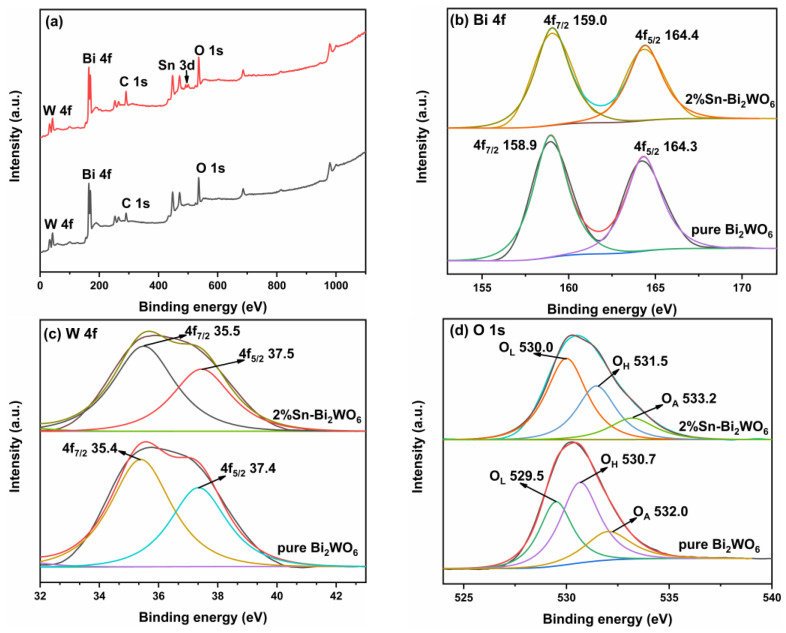

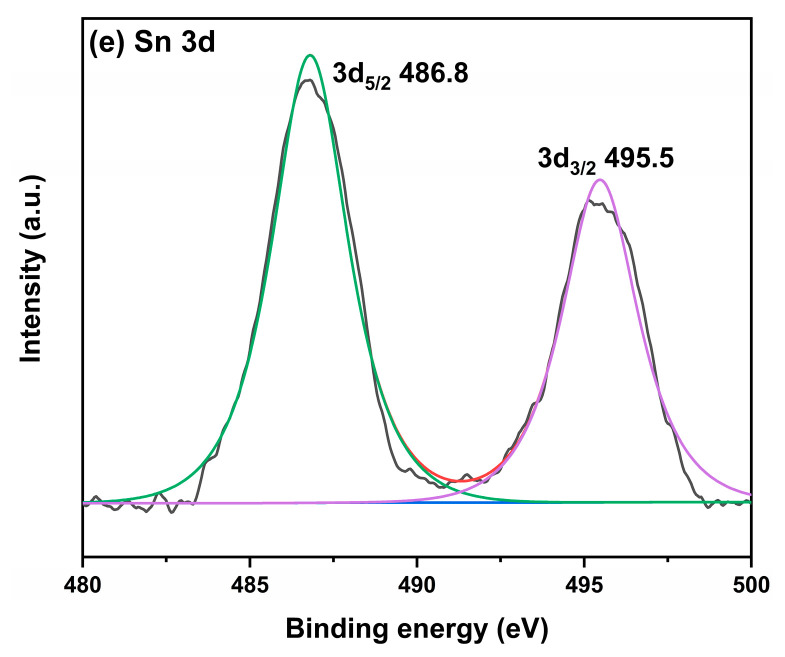
XPS survey of pure Bi_2_WO_6_ and 2%Sn-Bi_2_WO_6_ (**a**), high resolution spectra of Bi 4f (**b**), W 4f (**c**), O 1s (**d**), and Sn 3d (**e**).

Figure 5 presents the XPS spectra of pure Bi_2_WO_6_ and 2%Sn-Bi_2_WO_6_. The presence of signal peaks of Bi 4f, W 4f, O 1s, and C 1s in Bi_2_WO_6_ can be observed in Figure 5a. C element may be originated from the oil contamination [33]. The high-resolution spectra of Bi 4f are shown in Figure 5b. The spin-orbit of Bi 4f of pure Bi_2_WO_6_ splits into two characteristic peaks at 164.3 and 158.9 eV, corresponding to Bi 4f_5/2_ and Bi 4f_7/2_, indicating that the Bi element in the sample exists in the chemical state of Bi^3+^ [36,37,38]. The W 4f of pure Bi_2_WO_6_ has two characteristic peaks at 37.4 and 35.4 eV, corresponding to W 4f_5/2_ and W 4f_7/2_, verifying that the W element exists as the 6+ valence state [14,39,40]. The O 1s spectrum of pure Bi_2_WO_6_ (Figure 5d) is decomposed into three peaks at 529.5, 530.7, and 532.0 eV, corresponding to lattice oxygen (O_L_), surface hydroxyl (O_H_), and surface adsorbed oxygen (O_A_), respectively [41,42]. After Sn modification, the binding energies of Bi 4f, W 4f, and O 1s shift to a higher energy band, which can be ascribed to the interaction between Sn element and Bi, W, and O elements [38]. The Sn 3d shows two characteristic peaks at 486.8 and 495.5 eV, corresponding to Sn 3d_5/2_ and Sn 3d_3/2_, indicating that the Sn element exists in the chemical state of Sn^4+^ [28,43]. Figure S2 presents the XPS survey spectra of 1%Sn-Bi_2_WO_6_, 4%Sn-Bi_2_WO_6_, and 6%Sn-Bi_2_WO_6_. The binding energies of all the samples are shown in Table 2. Compared with pure Bi_2_WO_6_, the binding energies corresponding to the peaks of Bi 4f shift after Sn doping, which indicates that the electronic environment inside the Bi_2_WO_6_ lattice has changed, and proves that Sn^4+^ ions are incorporated into the Bi_2_WO_6_ lattice by doping [37,42].

**Figure 6:**
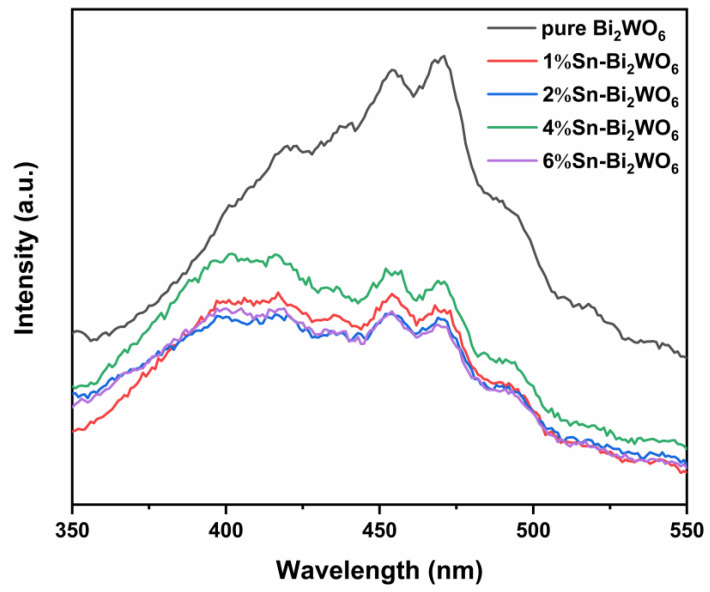
PL spectra of samples.

Figure 6 shows the PL spectra of samples. Sn-Bi_2_WO_6_ demonstrates lower intensity than pure Bi_2_WO_6_, indicating that Sn doping is beneficial to inhibiting the recombination of photogenerated electrons and holes. Crystal defects will be formed by Sn doping, which captures photogenerated charges, improving the separation of carriers [28,35]. The PL peak intensity of 2%Sn-Bi_2_WO_6_ is the lowest, indicating that the inhibition effect is the best when Sn/Bi molar ratio is 2%. The 4%Sn-Bi_2_WO_6_ showing a higher PL peak intensity than 2%Sn-Bi_2_WO_6_ can be ascribed to the fact that a high level of doping will bring new recombination centers, reducing the carrier separation [44]. The PL peak intensity of 6%Sn-Bi_2_WO_6_ is lower than 4%Sn-Bi_2_WO_6_. The XRD results prove that a new phase SnO_2_ appears in 6%Sn-Bi_2_WO_6_ and SnO_2_/Bi_2_WO_6_ semiconductor composite structure forms. The coupling of semiconductors with different energy band positions is beneficial to accelerating the migration of photogenerated carriers at the phase interfaces, improving the separation of photogenerated charges [39,45].

**Figure 7:**
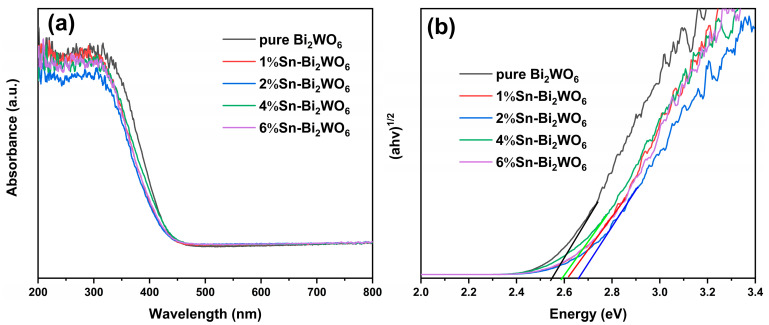
Diffuse reflectance spectra (**a**) and band gap energy (**b**) of samples.

The UV-visible absorption spectra of samples are shown in Figure 7a. The absorption edges of Sn-Bi_2_WO_6_ photocatalysts are similar, all around 440 nm, which is slightly smaller than that of pure Bi_2_WO_6_ (460 nm) [27]. It can be seen from Figure 7b that the band gap energies of pure Bi_2_WO_6_, 1%Sn-Bi_2_WO_6_, 2%Sn-Bi_2_WO_6_, 4%Sn-Bi_2_WO_6_, and 6%Sn-Bi_2_WO_6_ are 2.58, 2.62, 2.67, 2.59, and 2.62 eV, respectively.

### 2.2. Photocatalytic Performance

**Figure 8:**
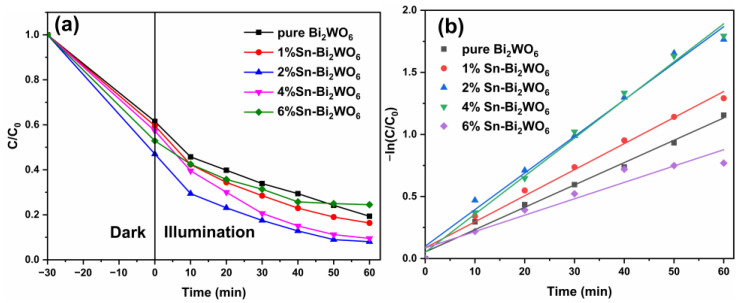
Photocatalytic degradation degree curves of MB (**a**) and kinetic fitting curves for pure Bi_2_WO_6_ and Sn-Bi_2_WO_6_ (**b**).

Figure 8a shows the degradation curves of MB by Sn-Bi_2_WO_6_ photocatalysts. The results show that the degradation degrees of 1%Sn-Bi_2_WO_6_, 2%Sn-Bi_2_WO_6_, and 4%Sn-Bi_2_WO_6_ are 83.7%, 92.0%, and 90.5%, respectively, which are higher than pure Bi_2_WO_6_ (80.6%) [27]. Sn doping improving the photocatalytic activity of Bi_2_WO_6_ can be attributed to the following two points: (1) The recombination of photogenerated charges is suppressed and the separation is improved by Sn doping. (2) The specific surface area of Bi_2_WO_6_ is enhanced after Sn doping, which is conducive to providing more active reaction sites, improving the photocatalytic efficiency [42]. It is worth noting that the degradation degrees of Sn-Bi_2_WO_6_ present a trend of first increasing and then decreasing, indicating that Sn doping level has an optimal doping concentration. The decreased photodegradation efficiency of 4%Sn-Bi_2_WO_6_ and 6%Sn-Bi_2_WO_6_ (75.5%) can be attributed to the formation of new recombination centers and excessive amorphous components due to excessive doping, respectively [28,45].

Figure 8b shows the first order kinetics curves of −ln(C/C_0_) versus time. The first-order reaction rate constants of pure Bi_2_WO_6_, 1%Sn-Bi_2_WO_6_, 2%Sn-Bi_2_WO_6_, 4%Sn-Bi_2_WO_6_, and 6%Sn-Bi_2_WO_6_ are 0.018, 0.021, 0.030, 0.031, and 0.013 min^−1^, respectively [27]. The first-order reaction rate constant of 2%Sn-Bi_2_WO_6_ is 1.7 times that of pure Bi_2_WO_6_.

**Figure 9:**
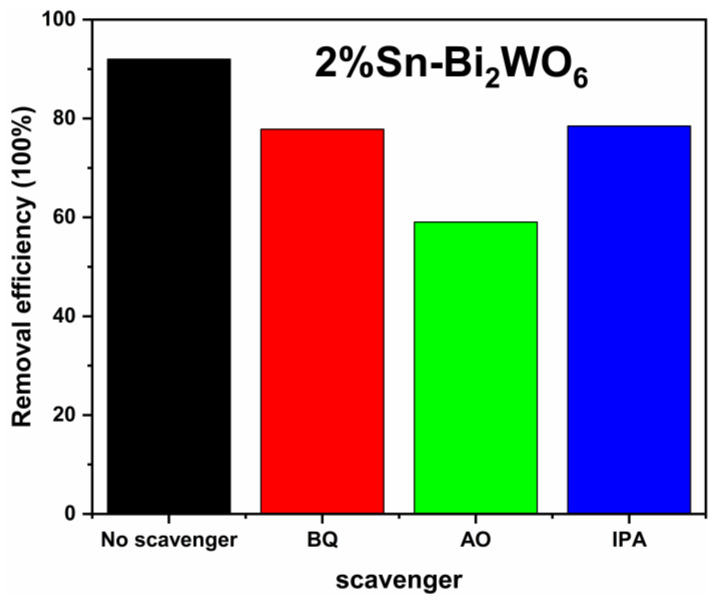
The degradation degrees of 2%Sn-Bi_2_WO_6_ in the presence of different scavengers.

Figure 9 shows the active species experiment of 2%Sn-Bi_2_WO_6_. During the experiments, holes (h^+^), ·O_2_^−^ and ·OH radicals could be quenched by benzoquinone (BQ), ammonium oxalate (AO), and isopropanol (IPA), respectively [25,41]. When BQ, AO, and IPA were added to the radical scavengers, the degradation degrees of 2%Sn-Bi_2_WO_6_ decreased from 92.0% to 77.8%, 59.0% and 78.6%, respectively. The results verify that holes (h^+^) are the main active species in the degradation process, and ·O_2_^−^ and ·OH radicals also contribute to the degradation.

According to Formulas (1) and (2), where E_VB_ is the valence band potential, E_CB_ is the conduction band potential, E_0_ is the free electron energy on the hydrogen scale (E_0_ = 4.5 eV); E_g_ is the bandgap energy of the photocatalytic material, and X is the absolute electronegativity of the semiconductor [46].

E_CB_ = X − E_0_ − E_g_/2 (1)

E_VB_ = E_g_ + E_CB_ (2)

**Figure 10:**
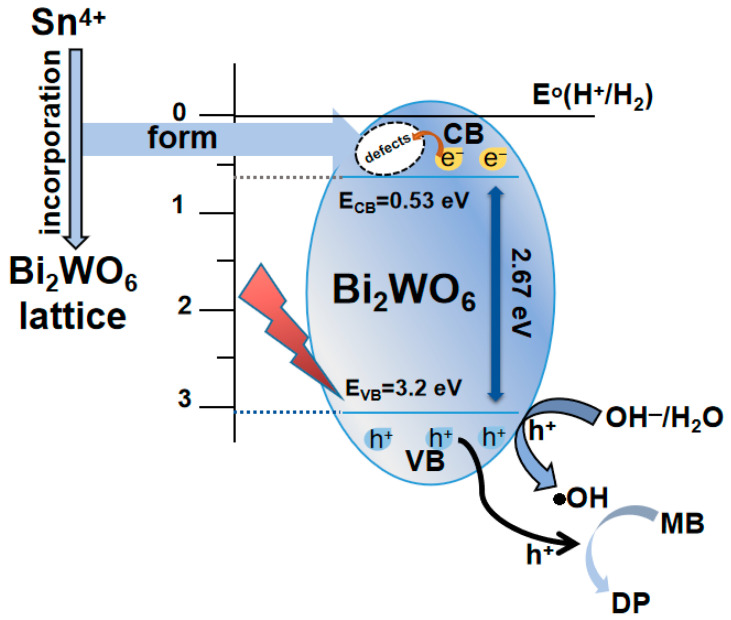
Schematic diagram of charge transfer and photodegradation of MB for 2%Sn-Bi_2_WO_6_.

The valence band potential (E_VB_) and conduction band potential (E_CB_) of 2%Sn-Bi_2_WO_6_ are 3.20 and 0.53 eV, respectively. Based on the results, a schematic diagram of the photodegradation MB by 2%Sn-Bi_2_WO_6_ is proposed, as shown in Figure 10. Sn doping introduces lattice distortion, forming more crystal defects, capturing photogenerated electrons, and preventing their recombination with holes. Consequently, the holes react with H_2_O/OH^−^ and generates strong oxidizing hydroxyl radicals (·OH). The holes and ·OH hydroxyl radicals directly decompose MB molecules into inorganic small molecules [47,48].

## 3. Materials and Methods

Bismuth nitrate (Bi(NO_3_)_3_·5H_2_O, Analytical Reagent, AR, ≥99.0%), sodium tungstate (Na_2_WO_4_·2H_2_O, AR, ≥99.5%), anhydrous ethanol (C_2_H_5_OH, AR, ≥99.7%), glacial acetic acid (C_2_H_4_O_2_, AR, ≥99.5%), and tin tetrachloride (SnCl_4_·5H_2_O, AR, ≥99.0%) were purchased from Chengdu Kelong Chemical Co., Ltd., Chengdu, China.

### 3.1. Sample Preparation

Bi_2_WO_6_: Bi(NO_3_)_3_·5H_2_O and CH_3_COOH were dispersed in 20 mL deionized water to form solution A, and disperse Na_2_WO_4_·2H_2_O in 12 mL deionized water to form solution B. The mass ratio of Bi(NO_3_)_3_·5H_2_O to Na_2_WO_4_·2H_2_O was 2.94:1. Solution B was added dropwise to solution A, and stirring was continued for 30 min to form white flocs. The resulting mixture was transformed to a polytetrafluoroethylene liner, put in a reaction kettle, tightened, and heated at 160 °C 24 h. Alternately it was washed with deionized water and absolute ethanol until neutral, placed in a drying oven at 100 °C for drying for 10 h, and finally fully ground to obtain pure Bi_2_WO_6_ powder.

X%Sn-Bi_2_WO_6_: SnCl_4_·5H_2_O was added to solution A, and other experimental conditions were consistent with the preparation of Bi_2_WO_6_, the Sn-doped Bi_2_WO_6_ photocatalytic material could be synthesized, and the molar ratio of Sn/Bi was controlled to be 1%, 2%, 4%, and 6%. The Sn-doped Bi_2_WO_6_ with different concentration gradients were labeled as X%Sn-Bi_2_WO_6_ (X = 1, 2, 4, 6).

### 3.2. Sample Characterization

The crystal structure and phase information were studied using X-ray diffraction (XRD) using a DX-2700 X-ray diffractometer with Cu Kα radiation as the X-ray source. The scan range 2θ was 20°–70° and scan speed was 0.06°/s (Dandong Haoyuan Instrument Co., Ltd., Dandong, China). The XRD data were analyzed using jade 6.0 software. FEI-nspect F50 scanning electron microscope (SEM) and FEI-Tecnai G2 F20 transmission electron microscope (TEM and HRTEM) were used to observe the morphology (FEI Company, Hillsboro, OR, USA); the specific surface area were measured using a V-sorb 2800S analyzer (BET) (Mike Instrument Company, Atlanta, GA, USA); the composition and valence of elements were analyzed using an XSAM800 multifunctional surface analysis system (XPS) (Thermo Scientific K-Alpha, Kratos Ltd., Manchester, UK); the photoluminescence (PL) spectra were measured using an F-4600 fluorescence spectrum analyzer with a Xe lamp at an excitation wavelength of 320 nm (Shimadzu Group Company, Kyoto, Japan); the optical absorption was tested using a UV-3600 ultraviolet–visible photometer (DRS) (Shimadzu Group Company, Kyoto, Japan).

### 3.3. Photocatalysis Experiment

In total, 100 mL (10 mg/L) MB aqueous solution and 0.025 g samples were mixed into a beaker, placed in a dark state, and stirred for 30 min to reach the equilibrium of adsorption and desorption, and then a 250 W xenon lamp was used as the light source. Sampling occurred every 10 min, the samples were centrifuged to collect the upper clear solution, its absorbance A was measured at λ = 664 nm, and the degradation degree was calculated using the formula (A_0_ − A_t_)/A_0_ × 100%, where A_0_ and A_t_ are the initial and absorbance at time.

On the basis of the MB degradation system, 2 mL (0.1 mol/L) of benzoquinone (BQ, ·O_2_^−^ trapping agent), ammonium oxalate (AO, h^+^ trapping agent), and isopropanol (IPA, ·OH trapping agent) were added to investigate the active species.

## 4. Conclusions

The pure and Sn-doped Bi_2_WO_6_ nanomaterials with different concentrations were prepared by hydrothermal method, and the effects of Sn doping on the structure and photocatalytic performance of Bi_2_WO_6_ were studied. The results show that Sn-Bi_2_WO_6_ exhibits 3D flower-like morphology, and the nanosheets are thinner than pure Bi_2_WO_6_, which greatly increases the specific surface area. Sn doping does not cause a significant red shift; however, it promotes the separation of photogenerated electron–hole pairs. The photocatalytic degradation results show that the photocatalytic activity of 2%Sn-Bi_2_WO_6_ is the highest, and the degradation degree of MB is 92.0% after illumination for 60 min, which is higher than that of pure Bi_2_WO_6_ (80.6%). The first-order reaction rate constant of 2%Sn-Bi_2_WO_6_ is 0.030 min^−^^1^, which is 1.7 times that of pure Bi_2_WO_6_.

## Figures and Tables

**Table 1:** The lattice parameters of samples.

Sample	Lattice Constant			Crystal Vol /Å3
	a/Å	b/Å	c/Å	
Pure-Bi_2_WO_6_	5.4872	16.3120	5.4407	486.98
1%Sn-Bi_2_WO_6_	5.4652	16.5365	5.4354	491.23
2%Sn-Bi_2_WO_6_	5.4430	16.5068	5.4339	488.22
4%Sn-Bi_2_WO_6_	5.5106	16.7287	5.3707	495.10
6%Sn-Bi_2_WO_6_	5.4518	16.4035	5.6174	502.36

**Table 2:** Binding energies of samples.

	Bi		W		O			Sn	
Sample	4f_7/2_	4f_5/2_	4f_7/2_	4f_5/2_	O_L_	O_H_	O_A_	3d_5/2_	3d_3/2_
Pure Bi_2_WO_6_	158.9	164.3	35.4	37.4	529.5	530.7	532.0		
1%Sn-Bi_2_WO_6_	158.9	164.3	35.4	37.4	529.8	531.0	532.9	486.6	495.3
2%Sn-Bi_2_WO_6_	159.0	164.4	35.5	37.5	530.0	531.5	533.2	486.8	495.5
4%Sn-Bi_2_WO_6_	159.2	164.6	35.7	37.6	530.2	531.7	533.4	487.0	495.7
6%Sn-Bi_2_WO_6_	159.3	164.7	35.8	37.7	530.3	531.8	533.4	487.1	495.9

## Data Availability

Not applicable.

## Supplementary Materials

The following supporting information can be downloaded at: https://www.mdpi.com/article/10.3390/ijms23158422/s1.
