# Supplementary material for: Synthesis of Tin-Doped Three-Dimensional Flower-like Bismuth Tungstate with Enhanced Photocatalytic Activity
Source: Int J Mol Sci. 2022 Jul 29;23(15):8422. doi: 10.3390/ijms23158422 (PMC9369453; doi:10.3390/ijms23158422)
Supplement: Supplementary file 1 [file ijms-23-08422-s001.zip › ijms-1805680-supplementary.pdf]

International Journal of *Molecular Sciences*

MDPI

Article

Synthesis of Tin-Doped Three-Dimensional Flower-like Bismuth Tungstate with Enhanced Photocatalytic Activity

Xiaodong Zhu [1], Fengqiu Qin [1], Xiuping Zhang [1], Yuanyuan Zhong [1], Juan Wang [1], Yu Jiao [2,*], Yuhao Luo [3] and Wei Feng [1,*]

1 School of Mechanical Engineering, Chengdu University, Chengdu 610106, China; mysumeiren@163.com (F.Q.); 18781235109@163.com (X.Z.); suzyy605@163.com (Y.Z.); wangjuan9760@163.com (J.W.)
2 School of Science, Xichang University, Xichang 615013, China
3 College of Materials and Chemistry & Chemiacl Engineering, Chengdu University of Technology, Chengdu 610051, China; lyh1609460550@163.com
* Correspondence: jiaoyu@xcc.edu.cn (Y.J.); fengwei@cdu.edu.cn (W.F.)

Citation: Zhu, X.; Qin, F.; Zhang, X.; Zhong, Y.; Wang, J.; Jiao, Y.; Luo, Y.; Feng, W. Synthesis of Tin-Doped Three-Dimensional Flower-like Bismuth Tungstate with Enhanced Photocatalytic Activity. *Int. J. Mol. Sci.* **2022**, *23*, 8422. https://doi.org/10.3390/ijms23158422

Academic Editor: Andrea Salis

Received: 22 June 2022
Accepted: 27 July 2022
Published: 29 July 2022

Publisher's Note: MDPI stays neutral with regard to jurisdictional claims in published maps and institutional affiliations.

Copyright: © 2022 by the authors. Licensee MDPI, Basel, Switzerland. This article is an open access article distributed under the terms and conditions of the Creative Commons Attribution (CC BY) license (https://creativecommons.org/licenses/by/4.0/).

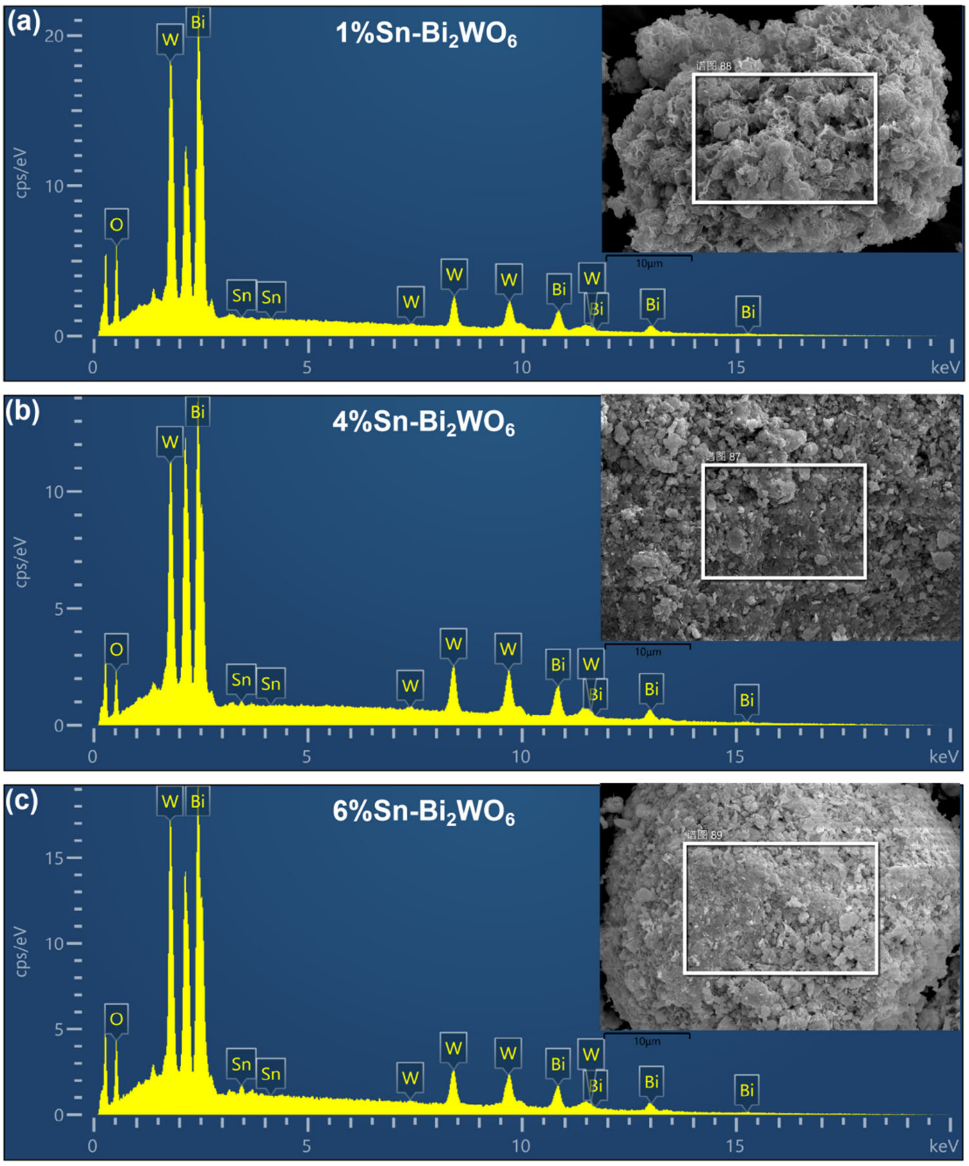

Figure S1. EDS analysis of 1%Sn-Bi_2WO_6 (a), 4%Sn-Bi_2WO_6 (b) and 6%Sn-Bi_2WO_6 (c).

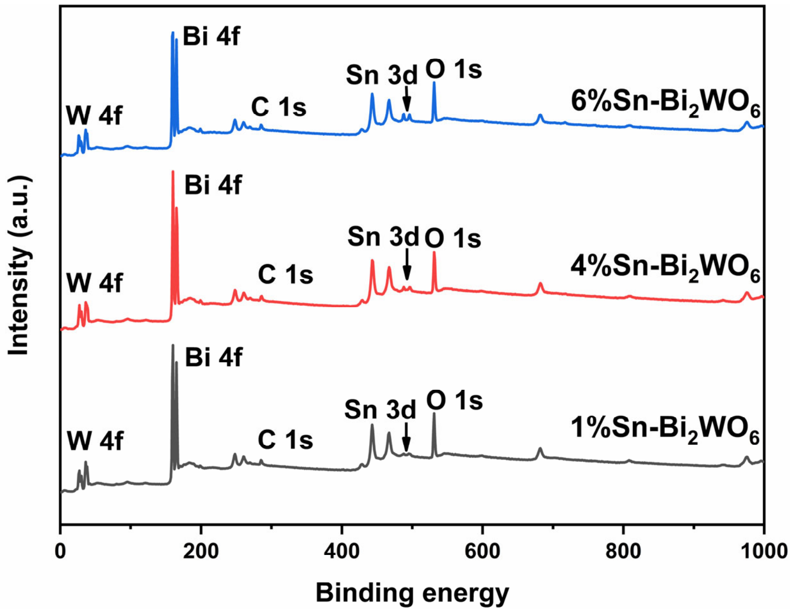

Figure S2. XPS survey spectra of 1%Sn-Bi_2WO_6, 4%Sn-Bi_2WO_6 and 6%Sn-Bi_2WO_6.
